# Resveratrol Protects PC12 Cell against 6-OHDA Damage via CXCR4 Signaling Pathway

**DOI:** 10.1155/2015/730121

**Published:** 2015-11-22

**Authors:** Jing Zhang, Wenchuang Fan, Hui Wang, Lihua Bao, Guibao Li, Tao Li, Shouyang Song, Hongyu Li, Jing Hao, Jinhao Sun

**Affiliations:** ^1^Department of Anatomy and Key Laboratory of The Ministry of Education for Experimental Teratology, School of Medicine, Shandong University, Jinan, Shandong 250012, China; ^2^Department of Traumatic Orthopaedics, Shandong Provincial Hospital, Shandong University, Jinan, Shandong 250012, China; ^3^Department of Neurosurgery, The Fourth Hospital of Jinan City, Jinan, Shandong 250012, China; ^4^Department of Histology and Embryology, School of Medicine, Shandong University, Jinan, Shandong 250012, China

## Abstract

Resveratrol, herbal nonflavonoid polyphenolic compound naturally derived from grapes, has long been acknowledged to possess extensive biological and pharmacological properties including antioxidant and anti-inflammatory ones and may exert a neuroprotective effect on neuronal damage in neurodegenerative diseases. However, the underlying molecular mechanisms remain undefined. In the present study, we intended to investigate the neuroprotective effects of resveratrol against 6-OHDA-induced neurotoxicity of PC12 cells and further explore the possible mechanisms involved. For this purpose, PC12 cells were exposed to 6-OHDA in the presence of resveratrol (0, 12.5, 25, and 50 *μ*M). The results showed that resveratrol increased cell viability, alleviated the MMP reduction, and reduced the number of apoptotic cells as measured by MTT assay, JC-1 staining, and Hoechst/PI double staining (all *p* < 0.01). Immunofluorescent staining and Western blotting revealed that resveratrol averts 6-OHDA induced CXCR4 upregulation (*p* < 0.01). Our results demonstrated that resveratrol could effectively protect PC12 cells from 6-OHDA-induced oxidative stress and apoptosis via CXCR4 signaling pathway.

## 1. Introduction

Parkinson's disease (PD) is a neurodegenerative disorder characterized by prominent selective loss of dopaminergic neurons in the substantia nigra (SN) and other parts of the brain, which mainly affects elder persons. It is now widely accepted that the classical symptoms of PD are the occurrence of rigidity, tremor, bradykinesia, and hypokinesia [1, 2]. Besides, there is ample evidence that PD often goes with nonmotor symptoms, like sleep disturbances, anosmia, cognitive decline, and psychiatric disorders. These symptoms appear in the early stages of PD constantly and could not be effectively attenuated by conventional anti-Parkinsonian medications [3–5]. Increasing evidences have shown that PD may be associated with mitochondrial dysfunction, oxidative stress, inflammation, glutamatergic toxicity or protein misfolding, and aggregation [6–8]. Additionally, mitochondrial dysfunction, increased oxidative stress, and inflammation may lead to apoptosis and necrosis of neurons and are involved in neurodegeneration [9, 10]. Although great advances have been achieved in the etiology of this disease, the causes of the selective degeneration of dopaminergic neurons and the molecular mechanisms controlling these events are largely unclear.

As a nonflavonoid polyphenolic compound abundant in many plant species, such as grapes, mulberries, peanuts, and red wines, resveratrol possesses many biological functions such as inhibiting phenomena associated with inflammatory, aging, oxidant, and cancer [11–14]. Of late, many studies evaluated resveratrol as a protective factor against different kinds of neurotoxin, axonal degeneration, and neurodegenerative diseases [15]. Our previous studies also have described that resveratrol exerted neuroprotective effects against A*β*-induced neurotoxicity and inhibited cell apoptosis [16, 17]. Besides, resveratrol could slow down cognitive decline and participate in cell signalling modulation, antiamyloidogenic activity, modulation of telomere length, and the sirtuin proteins [18]. Khan et al. have reported that resveratrol demonstrated neuroprotective effects against 6-OHDA-induced oxidative damage and dopamine depletion in rat model of Parkinson's disease [19]. Therefore, great deal of work has been done about the neuroprotective properties of resveratrol, but whether resveratol has protective effects on cultured PC12 cells against 6-OHDA-induced damage and if so, what the underlying mechanisms have not been fully understood.

Since autoimmunity and inflammatory response can be a source of injury to neurons [20] and activated microglia may participate in the neuronal damage in PD [21, 22], great effort has been done centered on inflammation and PD. But the role of chemokines and their receptors in PD is less understood. Chemokines are small, secreted proteins whose function is attracting immune cells migrating into inflammatory sites and secondary lymphoid organs [23]. CXCR4, a specific G-protein coupled seven-transmembrane span receptor, is one of the most common chemokine receptors, whose natural ligand is CXCL12 (also known as SDF-1). CXCR4 plays a crucial role in the development of the nervous system [24]. In the central nervous system (CNS), CXCR4 participated in axonal growth, intercellular communications, neuromodulation, and mediation of signal transduction [25, 26]. It may also be implicated in neuroinflammatory and neurodegenerative disorders, including multiple sclerosis [27], human immunodeficiency virus-1 (HIV) encephalitis [28], and Alzheimer's disease [29]. It has been reported that, in rodents, the nigrostriatal system exhibits high expression of CXCL12 and CXCR4 [30] to modulate DA transmission and promote neuronal apoptosis [31]. With a MPTP model of PD, Shimoji et al. found that MPTP could induce a time-dependent upregulation of CXCR4 and this upregulation occurred before the loss of dopaminergic neurons and demonstrated that CXCL12/CXCR4 may be implicated in the etiology of PD [31]. Thus, we suppose that resveratrol may prevent the damage induced by 6-OHDA through affecting CXCR4 signaling.

In this current study, we investigated the protective effects of resveratrol on a neurotoxic cell model of 6-OHDA injury using PC12 cells. MTT assay was performed to determine the effects of resveratrol on this 6-OHDA-induced damage. Cell apoptosis was observed by Hoechst 3342/PI double staining. JC-1 mitochondrial dye was used to assess changes in the mitochondrial membrane potential. Also, immunofluorescent assay, real-time PCR, and Western blotting were applied to determine whether resveratrol offers neuroprotection via CXCR4 signaling pathway.

## 2. Materials and Methods

### 2.1. Materials

High differentiated PC12 cell line was obtained from the Cell Bank of the Chinese Academy of Sciences (Shanghai, China). 6-Hydroxydopamine (6-OHDA), resveratrol (3,4,5′-trihydroxy-trans-stilbene), dimethyl sulfoxide (DMSO), and MTT assay kit were provided by Sigma-Aldrich Inc. (St. Louis, MO, USA). Dulbecco's modified Eagle's medium (DMEM) and fetal bovine serum (FBS) were purchased from Hyclone Company (Logan, UT, USA). Polyclonal rabbit anti-CXCR4 antibody was purchased from Abcam (Cambridge, Massachusetts, USA). All other chemicals were obtained from commercial sources.

### 2.2. Culture and Treatment of Cells

PC12 cells were cultured in DMEM supplied with 10% FBS at 37°C, in 5% CO_2_ humidified incubator, and the medium was refreshed every three days. Before initiating 6-OHDA treatment, cells were seeded in culture plates at a density of 2 × 10^4^ cells/cm^2^ and cultured at 37°C for at least 24 h and then incubated with 6-OHDA at different concentrations (25, 50, 100, and 150 *μ*M). 24 h after the exposure to 6-OHDA, cells were examined under an inverted microscope.

For protection experiments of resveratrol, cells were separated randomly into three groups and treated (1) with 50 *μ*M 6-OHDA in 6-OHDA injury group, (2) with 12.5, 25, and 50 *μ*M resveratrol 2 h prior to treatment with 50 *μ*M 6-OHDA in resveratrol protection group, and (3) with nothing in normal control group. 24 h later, cells in all groups were collected and proceeded to the subsequent assays.

### 2.3. Cell Viability Assay

Cell viability was determined using MTT assay. Briefly, PC12 cells were cultured in 96-well plates at a density of 2 × 10^5^ cells/cm^2^. After being exposed to different drugs for 24 h, the cells were incubated with 0.5 mg/mL MTT at 37°C for 4 h. Then, to dissolve the formazan crystals formed, 100 *μ*L of DMSO was added. A microplate reader (Multiskan MK3, Thermo Labsystems, Philadelphia, PA, USA) was used to quantify the absorbance of each well at 490 nm (A490). The cells viability was indicated by the optical density (OD) of this absorbance.

### 2.4. Hoechst 33342/Propidium Iodide (PI) Double Staining

Hoechst 33342/PI double staining was performed to evaluate the protective effects of resveratrol against 6-OHDA induced apoptosis. Briefly, PC12 cells in each group were stained with Hoechst 33342 (10 *μ*g/mL) followed by PI (10 *μ*g/mL) for 15 min at 37°C, respectively. Then the labeled cells were observed with a 780 laser scanning confocal microscope (Carl Zeiss SAS, Jena, Germany).

### 2.5. 5,5′,6,6′-Tetrachloro-1,1′,3,3′-tetraethylbenzimidazolcarbocyanine Iodide (JC-1) Staining

Changes of the mitochondrial membrane potential (MMP) were measured using JC-1 staining (Invitrogen, USA) according to the manufacturer's instructions. 24 h after treatment, PC12 cells in each group were incubated with 10 *μ*M JC-1 dye at 37°C for 30 min. After this, cells were washed with PBS twice and observed under a Zeiss 780 laser scanning confocal microscope (Carl Zeiss SAS, Jena, Germany), with excitation wavelength at 488 nm and emission wavelength at 527 nm and 590 nm. 

### 2.6. Immunofluorescent Staining

To analyze the expression of CXCR4, immunofluorescent staining was performed. Briefly, PC12 cells in each group were fixed with 4% paraformaldehyde for 10 min at room temperature followed by permeabilizing using 0.2% Triton X-100 in PBS. Then the cells were incubated with 10% normal goat serum for 60 min to block nonspecific antibody binding. Subsequently primary antibody against CXCR4 (diluted 1 : 200) was added, and the cells were incubated overnight at 4°C. After washing with PBS for 3 times, PC12 cells were incubated with secondary antibodies (tetramethylrhodamine isothiocyanate labeled goat anti-rabbit IgG, diluted 1 : 200, Zhongshan Golden Bridge Biotechnology, Beijing, China). The stained cells were observed with a fluorescent inverted phase-contrast microscope (IX70, Olympus, Japan).

### 2.7. Western Blotting

PC12 cells were collected, washed with PBS, and lysed using RIPA lysis buffer (Beyotime Institute of Biotechnology, Shanghai, China). Then the protein content was measured using BCA kit (Boster Biological Technology, Wuhan, China) to determine protein concentration. After being separated by 10% sodium dodecyl sulfate (SDS) polyacrylamide gel/Tris-glycine electrophoresis, the proteins were transferred electrophoretically onto nitrocellulose (NC) membrane. The membrane was subsequently blocked with 5% defatted milk in Tris-buffer containing 0.1% Tween-20 and then incubated with primary anti-CXCR4 (1 : 1,000) antibody at 4°C overnight. Afterwards, the membrane was incubated with HRP labeled secondary IgG (1 : 1,000, ZSGB-BIO ORIGENE, Beijing, China) at room temperature for 1 h. Then the bands were developed with ECL reagent (Millipore Corporation, Billerica, MA, USA) and analyzed using Image J software (National Institute of Health, USA).

### 2.8. Effect of AMD3100 on 6-OHDA's Damage

AMD3100 (Sigma, St. Louis, MO), a specific inhibitor of CXCR4, was used to evaluate the role of CXCR4 in 6-OHDA induced neuron injury. 24 h after* in vitro* culture, PC12 cells were treated with AMD3100 with a final concentration of 10 mg/mL as previously reported [32] for 5 min before 50 *μ*M 6-OHDA damage. The cell growth was determined by MTT assay, and the cell apoptosis was evaluated by Hoechst 33342/PI double staining.

### 2.9. Statistical Analysis

All experiments were repeated three or more times. All the data in this study were expressed as mean ± S.E.M. Statistical significance was processed with analysis of variance (ANOVA) followed by the Limited Slip Differential (LSD) post hoc tests using the SPSS 17.0 software. *p* < 0.05 was regarded as statistically significant.

## 3. Results

### 3.1. Neurotoxicity Induced by 6-OHDA

**Figure 1:**
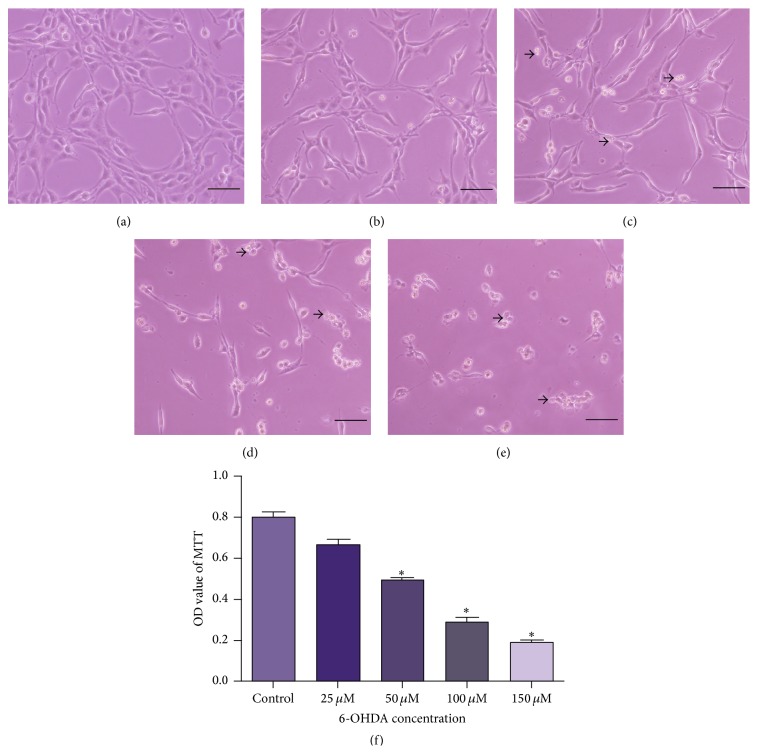
6-OHDA induced damage to the cultured PC12 cells. (a–e) The photomicrographs of PC12 cells exposed to 6-OHDA of different concentration for 24 h. (a) PC12 cells in routine culture medium grew well with long neurites. (b) When exposed to 25 *μ*M 6-OHDA, cell morphology had little changes. (c) When treated with 50 *μ*M 6-OHDA, the neurites started to retract, and the neural network was collapsed in a certain degree with cell debris that appeared. (d and e) As the concentration of 6-OHDA increased to 100 and 150 *μ*M, the cells were strongly insulted and severe cell loss could be observed. There was a mass of cell debris that could be found in the medium. Arrows indicated cell debris. (f) MTT assay also demonstrated that cell viability decreased gradually with the concentration of 6-OHDA increased. OD values were presented as means ± SD from five independent experiments. ^*∗*^ *p* < 0.01 versus control group. Scale bars: (a–e) 100 *μ*m.

To establish the neurotoxic cell model with 6-OHDA, PC12 cells were treated with 6-OHDA of different concentrations (25, 50, 100, and 150 *μ*M). As shown in Figure 1, exposure to 6-OHDA could induce damage of varying degrees on PC12 cells. In routine culture medium, PC12 cells grew in good condition with long neurites (Figure 1(a)). When the cells were treated with 25 *μ*M 6-OHDA, there were few changes on cell morphology (Figure 1(b)). As the concentration of 6-OHDA increased to 50 *μ*M, the cell number decreased, cells began to swell, the neurites started to retract, the network was collapsed, and cell debris appeared (Figure 1(c)). While being exposed to 100 and 150 *μ*M 6-OHDA, PC12 cells were strongly insulted, the cell number was largely decreased, and cell debris could be observed easily (Figures 1(d) and 1(e)). When incubated with 150 *μ*M 6-OHDA especially, almost all the neurites were lysed and a mass of cell debris was present. Consistent with morphological observations, MTT assay further demonstrated that, with the concentration of 6-OHDA increased, cell viability decreased gradually (Figure 1(f)). Therefore, 50 *μ*M 6-OHDA was selected to induce cell damage in the following experiments.

### 3.2. Beneficial Effect of Resveratrol on 6-OHDA Induced Neurotoxicity

**Figure 2:**
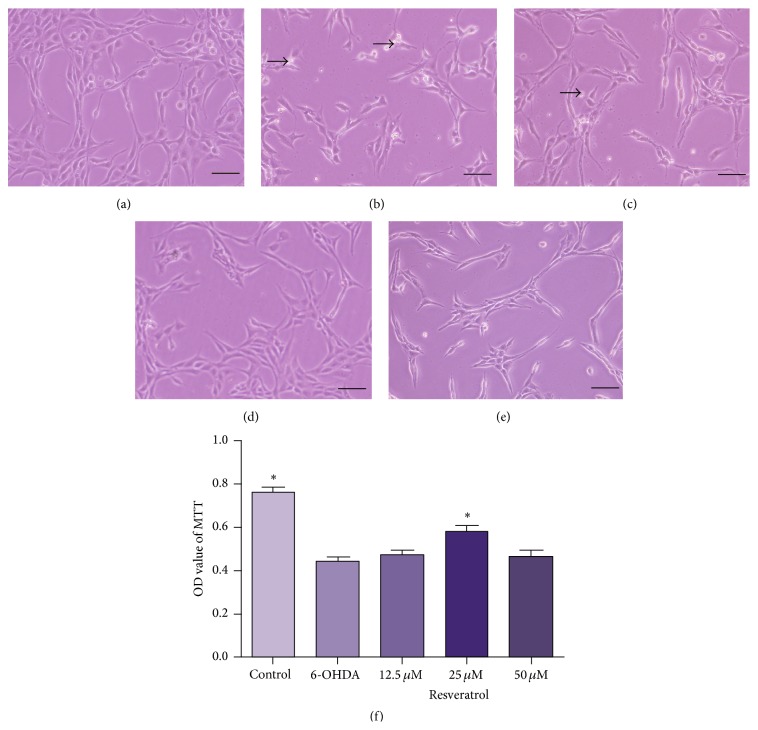
Protection of resveratrol on PC12 cells against 6-OHDA. (a) PC12 cells of normal control group grew in good condition and exhibited long neurites. (b) In 6-OHDA injury group, neurites were short and few with the neural network collapsed. (c–e) The cells were preincubated for 2 h with resveratrol at 12.5 (c), 25 (d), and 50 *μ*M (e) before 50 *μ*M 6-OHDA exposure, respectively. In 12.5 *μ*M resveratrol group, the protective effect was weak with swollen cells and cell debris observed (c). 25 *μ*M resveratrol promoted neurite growth, and only little cell debris could be found (d). However, in 50 *μ*M resveratrol group, the alleviative effect was not obvious, and some cells swelled and turned into a round shape (e). Arrows (b and c) indicated cell debris. Cell viability was further measured by MTT assays (f). OD values were presented as means ± SD from five independent experiments. ^*∗*^ *p* < 0.01 versus 6-OHDA injury group. Scale bars: (a–d) 100 *μ*m.

To investigate whether resveratrol has protective impact against 6-OHDA-induced neuron damage, PC12 cells were preincubated for 2 h with resveratrol of different concentrations (12.5, 25, and 50 *μ*M), before the addition of 50 *μ*M 6-OHDA (Figure 2). Compared with the cells exposed to 6-OHDA alone (Figure 2(b)), 12.5 *μ*M resveratrol exhibited a weak protective effect (Figure 2(c)), while 25 *μ*M resveratrol effectively increased the cell viability, with neurite growth obviously promoted and cell debris reduced largely (Figure 2(d)). However, when preincubated with 50 *μ*M resveratrol, the alleviative effect was not obvious, and some cells swelled and turned into a round shape (Figure 2(e)). The results of MTT assay (Figure 2(f)) further confirmed the protective impact against 6-OHDA-induced cell damage. The A490 values in each resveratrol protection group were higher than those in 6-OHDA injury group, and the OD value of 25 *μ*M resveratrol was the highest (*p* < 0.01).

### 3.3. Antiapoptosis Effects of Resveratrol in 6-OHDA

**Figure 3:**
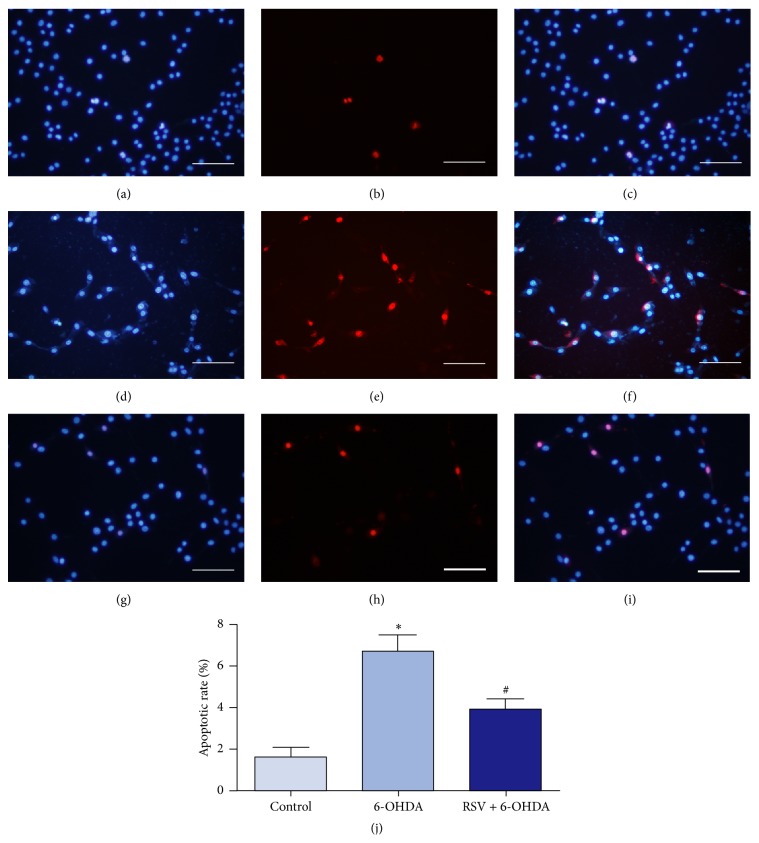
Resveratrol prevents 6-OHDA-induced cell apoptosis. PC12 cells were exposed to 6-OHDA with or without resveratrol for 24 h and double stained with Hoechst 33342 (blue) and PI (red) to determine cell apoptosis. In the control group, PC12 cells were cultured in DMEM, and most of the cells displayed normal nuclear morphology with uniform blue nuclei (a–c). In 6-OHDA injury group, the numbers of cells with bright red nuclei were obviously increased (d–f). And only few apoptotic cells could be observed when preincubated with 25 *μ*M resveratrol (g–i). (j) Statistical analysis showed that, compared with the control group, PC12 cells treated with 6-OHDA exhibited greater apoptotic rate (^*∗*^ *p* < 0.01, *n* = 5), while when preincubated with 25 *μ*M resveratrol, the apoptotic rate was largely decreased (^#^ *p* < 0.01, *n* = 5). Scale bars: (a–i) 100 *μ*m.

Hoechst 33342/PI double staining was performed to evaluate the effects of resveratrol on 6-OHDA induced apoptosis. Hoechst 33342 can stain living cells with a blue fluorescence, while PI can only permeate to damaged cell membrane and exhibit a red fluorescence. Therefore, survival cells displayed bright blue integrated nuclei, while the apoptotic cells were stained with bright red fragmented nuclei (Figure 3). As shown in Figures 3(a)–3(c), most of the cells in the normal control group had normal nuclear morphology with uniform blue nuclei. When exposed to 50 *μ*M 6-OHDA, much more cells could be observed with bright red nuclei (Figures 3(d)–3(f)). By contrast, only a few apoptotic cells were observed in resveratrol protection groups (Figures 3(g)–3(i)). Statistical analysis showed that PC12 cells exposed to 6-OHDA exhibited greater apoptotic rate than the control group (*p* < 0.01) and preincubation with resveratrol could definitely decrease the cell apoptosis induced by 6-OHDA (Figure 3(j), *p* < 0.01).

### 3.4. Resveratrol Alleviates 6-OHDA-Induced Changes of Mitochondrial Membrane Potential

**Figure 4:**
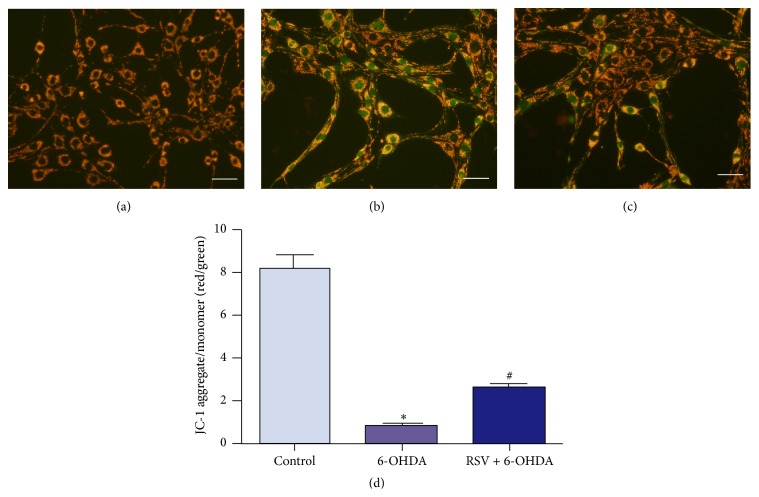
Effects of 6-OHDA and resveratrol on MMP of PC12 cells. (a–c) The fluorescent images of PC12 cells stained with JC-1 dye and captured by a Zeiss 780 laser confocal microscope. In the normal control group, cells exhibited clearly orange red (a), while, in 6-OHDA injury group, quite amounts of cells had high level of green fluorescent intensity, with lower level of red fluorescence (b). When preincubated with resveratrol, the green fluorescent intensity was effectively decreased meanwhile red fluorescence was increased (c). (d) Quantification of fluorescent intensity of PC12 cells in different group. Statistical analysis showed that the red/green fluorescence intensity ratio of PC12 cells treated with 6-OHDA was obviously decreased, which could be prevented by 25 *μ*M resveratrol. Ratio of red/green fluorescence was presented as means ± SD from five independent experiments. ^*∗*^ *p* < 0.01 versus control group, ^#^ *p* < 0.01 versus 6-OHDA injury group. Scale bars: (a–c) 50 *μ*m.

As an important determinant of early apoptosis, MMP was measured using JC-1 staining. In living cells, JC-1 is aggregated in mitochondria and emits red fluorescence, while, in apoptotic cells, JC-1 exists as a green fluorescence monomer and accrues in the cytosol. The ratio of red fluorescence to green fluorescence could reflect the intensity of MMP [33]. As shown in Figure 4(a), in the normal control group, cells clearly appeared orange red. After being treated with 6-OHDA, the green fluorescence was much brighter, and red fluorescence was decreased, indicating MMP decrease (Figure 4(b)). In the presence of resveratrol, the green fluorescent intensity was effectively decreased meanwhile red fluorescence was increased (Figure 4(c)). Statistical analysis showed that PC12 cells treated with 6-OHDA exhibited obviously decreased red/green fluorescent intensity ratio (*p* < 0.01), which could be prevented by 25 *μ*M resveratrol (Figure 4(d), *p* < 0.01). These results suggested that the mitochondrial pathway was involved in resveratrol's neuronal protective effect against 6-OHDA-induced cell apoptosis.

### 3.5. Resveratrol Downregulates the Expression of CXCR4 in PC12 Cells Treated with 6-OHDA

**Figure 5:**
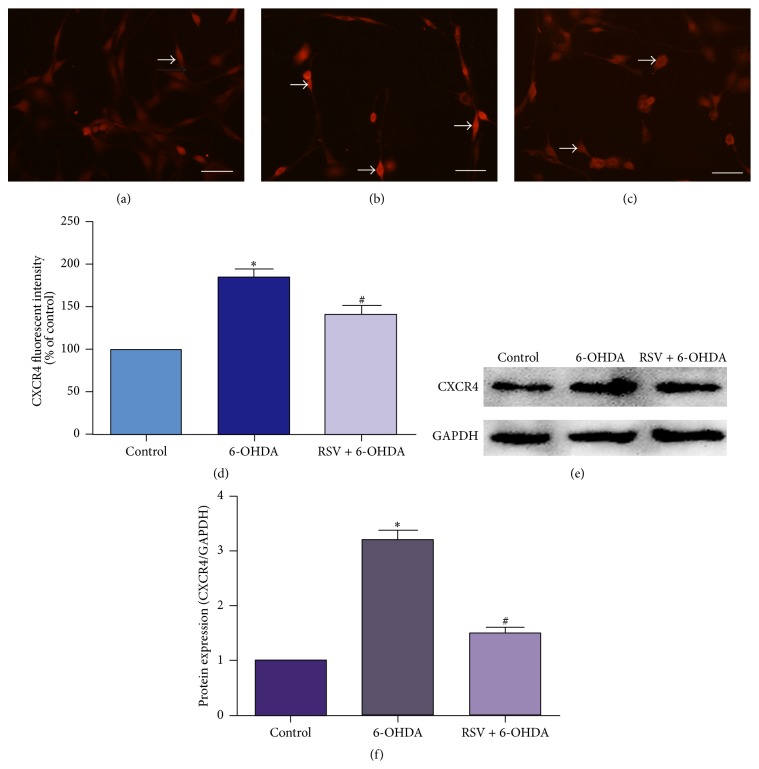
Resveratrol downregulates the expression of CXCR4 in PC12 cells treated with 6-OHDA. Immunofluorescent staining (a–d) and Western blotting (e, f) were used to determine the expression of CXCR4. (a) In normal control group, the intensity of the staining was weak. After being treated with 6-OHDA, the fluorescent intensity was increased (b). In the presence of resveratrol, this increase was effectively decreased (c). Arrows (a, b, and c) indicated the positive fluorescent staining. (d) Quantification of fluorescent intensity of PC12 cells in different group. In Western blotting, the level of CXCR4 protein was significantly increased in cells treated with 6-OHDA and preincubation with resveratrol could decrease the upregulated protein levels of CXCR4 (e and f). Values of fluorescent intensity were presented as means ± SD from five independent experiments. ^*∗*^ *p* < 0.01 versus control group, ^#^ *p* < 0.01 versus 6-OHDA injury group. Scale bars: (a–c) 100 *μ*m.

To provide a clue as to whether CXCR4 could be involved in the protective mechanisms of resveratrol against 6-OHDA-induced neuron injury, we measured the expression level of CXCR4 in PC12 cells treated with 6-OHDA after being preincubated with or without resveratrol through immunofluorescent staining and Western blotting. In normal control group, the intensity of the staining was weak, indicating that the expression of CXCR4 was low (Figure 5(a)). After being treated with 6-OHDA, the expression of CXCR4 was upregulated, as shown by the increased fluorescent intensity (Figure 5(b)). With the increase of the concentration of 6-OHDA, CXCR4 fluorescent intensity was increased gradually (date is not shown). However, preincubation with resveratrol could evidently prevent these upregulation processes (Figures 5(c) and 5(d), *p* < 0.01). Consistently, Western blotting also demonstrated that the level of CXCR4 protein was significantly increased in PC12 cells treated with 6-OHDA and this increase was prevented by preincubation with resveratrol (Figures 5(e) and 5(f), *p* < 0.01).

### 3.6. AMD3100 Protected PC12 Cells from 6-OHDA Induced Damage

**Figure 6:**
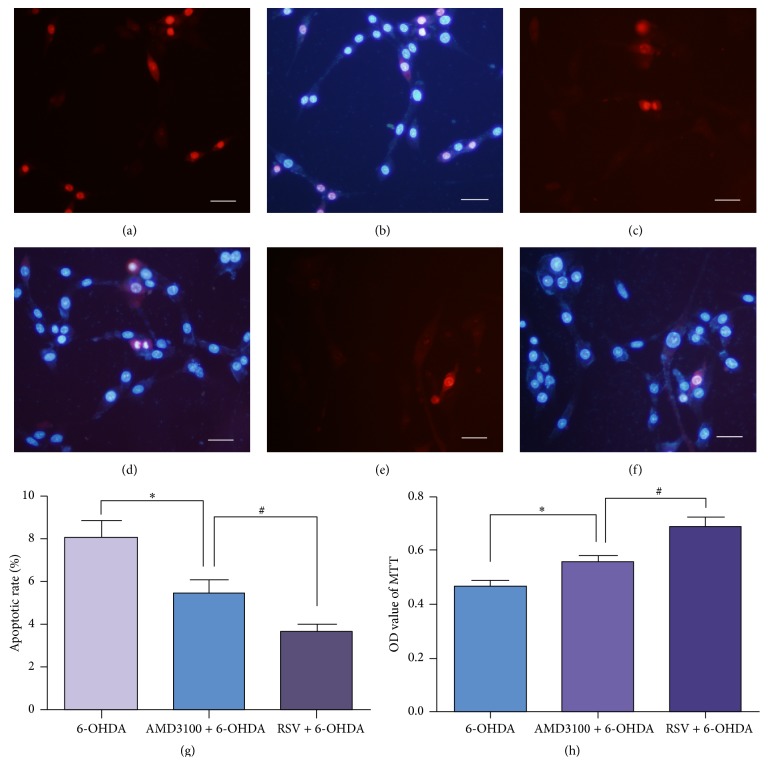
CXCR4 inhibitor AMD3100 protected PC12 cells from 6-OHDA induced damage. To further investigate the role of CXCR4 in 6-OHDA induced neurotoxicity, AMD3100 was added into the culture medium before 6-OHDA treated. Cell apoptosis was detected by Hoechst 33342 (blue) and PI (red) double staining (a–f) and observed under Zeiss LSM 780 laser confocal microscope. The number of apoptotic cells in AMD3100 group (c, d) was less than that in 6-OHDA group (a, b) but still more than that in resveratrol protection group (e, f). Cell apoptosis analysis (g) and MTT assay (h) demonstrated that AMD3100 could protect PC12 cells from injury induced by 6-OHDA. Data were presented as means ± SD. ^*∗*^ *p* < 0.05 versus 6-OHDA injury group, ^#^ *p* < 0.05 versus resveratrol protection group (*n* = 3). Scale bars: (a–f) 50 *μ*m.

As we found CXCR4 expression was upregulated with 6-OHDA exposure, the role of CXCR4 in 6-OHDA induced neurotoxicity was investigated by adding AMD3100, a specific inhibitor of CXCR4, into the culture medium before 6-OHDA exposure. Hoechst 33342/PI double staining showed that, compared with 6-OHDA group (Figures 6(a) and 6(b)), less apoptotic cells could be observed when pretreated with AMD3100 (Figures 6(c) and 6(d)), while the apoptotic cells were still more than that in resveratrol protection group (Figures 6(e) and 6(f)). Statistical analysis demonstrated that the apoptotic rate in the AMD3100 group was lower than that in the 6-OHDA injure group but still higher than the apoptotic rate in resveratrol protection group (Figure 6(g), *p* < 0.05). MTT assay also confirmed that being preincubated with AMD3100 could increase the cell viability which was decreased by 6-OHDA exposure (Figure 6(h), *p* < 0.05), though the cell viability in AMD3100 group remained lower than that in resveratrol protection group. Therefore, CXCR4 may participate in the protective process of resveratrol against 6-OHDA neurotoxicity.

## 4. Discussion

In the present study, an* in vitro* 6-OHDA damaged model of PC12 cells was used to investigate the protective effects of resveratrol against 6-OHDA neurotoxicity. Our data showed that resveratrol could significantly alleviate the MMP reduction, attenuate neuron apoptosis, increase cell viability, and thus effectively protect PC12 cells from 6-OHDA induced oxidative stress and apoptosis. Furthermore, we explored the underlying neuroprotective mechanisms and found that resveratrol prevented 6-OHDA induced upregulation of CXCR4 expression, indicating that CXCR4 signaling pathway may participate in the neuroprotective effects of resveratrol against the neurotoxicity of 6-OHDA.

Recent studies have suggested that resveratrol has neuroprotective effects against several kinds of injury [15, 17, 19, 34]. Resveratrol can ameliorate the cognitive degeneration, enhance mitochondrial oxidative function, inhibit reactive oxygen species (ROS) generation, increase autophagolysosome formation, and regulate apoptosis in several pathophysiologic processes [35–38]. In our present study, we also measured the protective effects of resveratrol on PC12 cells injured by 6-OHDA and confirmed that resveratrol significantly retarded neurotoxicity induced by 6-OHDA. In the presence of 6-OHDA, the viability of PC12 cells decreased gradually with 6-OHDA concentration increasing from 25 *μ*M to 150 *μ*M. But pretreatment with resveratrol apparently prevented this trend. In agreement with previous reports [39], our data strongly suggest that the neuroprotective effects of resveratrol depended on concentration used. When preincubated with low concentration (12.5 *μ*M) of resveratrol, PC12 cells still exhibited a certain degree of damage after being treated with 6-OHDA, while, given higher concentration (25 *μ*M) of resveratrol, cell condition was much better, with promoted neurite growth and decreased cell debris. It was noticed that resveratrol could increase MMP and attenuate 6-OHDA-induced cell apoptosis. The mitochondrial function increase and apoptosis-inhibitory effects of resveratrol have also been reported by many researches [8, 38]. Resveratrol could attenuate methylglyoxal-induced mitochondrial dysfunction and apoptosis [40]. Besides, resveratrol also could upregulate the antioxidant defenses and decrease the dopamine loss in PD model [19]. Hence, a certain concentration of extrinsic resveratrol administration significantly increased the viability of neuronal cells, protected them from cell death, and may be useful to reduce the neurons loss that occurs during PD.

Another interesting correlation among resveratrol and neuronal degeneration derives from experimental data showing that resveratrol suppresses the inflammatory responses and protects PC12 cells from inflammation-mediated damage [41, 42]. Autoimmunity and inflammatory responses can ultimately cause the damage of neurons in PD. Meanwhile chemokine CXCL12 and its receptor CXCR4 participate in the selective loss of DA neurons in this cascade of events [31, 43]. There are numerous evidences suggesting that CXCR4 signaling may cause neurodegeneration [27, 29, 44]. Thus we detected CXCR4 expression in PC12 cells treated with 6-OHDA after being preincubated with or without resveratrol through immunofluorescent staining and Western blotting. Our results showed that 6-OHDA upregulated CXCR4 expression, while resveratrol decreases its expression. When given AMD3100 to block CXCR4 pathway, the decreased cell viability and increased cell apoptotic rate induced by 6-OHDA remitted. It indicated that, at least partly, CXCR4 signal pathway was involved in resveratrol's neuroprotective effects against 6-OHDA's injury. CXCR4 has shown neurotoxicity to various neurons through the release of neurotoxins from microglia [45] or by a direct mechanism [31, 46]. We supposed that 6-OHDA activated CXCR4 and through some direct or indirect action to induce PC12 cell death, while preincubation with resveratrol can downregulate CXCR4's expression and interfere this process and play a protective role.

Although the exact pathogenesis of the selective neuronal loss in PD is largely unknown, many lines of evidence have proved that mitochondrial dysfunction and oxidative stress may play a crucial role [47, 48]. In postmortem PD striatum and substantia nigra, obvious oxidative damage to lipids, proteins, and DNA had been observed [49]. 6-OHDA, a neurotoxin been used to establish an experimental animal model of PD, can utilize the catecholamine transporter system to enter into dopaminergic neurons and generates H_2_O_2_ from its autooxidation reaction with oxygen to induce selective dopaminergic neuronal loss in the SN [50]. In this study, we observed the effect of 6-OHDA on PC12 cells and confirmed that exposure to 6-OHDA could induce neurites retraction, network collapse, and cell MMP decrease and increase the apoptosis of PC12 cells. It is well known that the changes of MMP could present mitochondrial function. Mitochondrial dysfunction is characterized by an increase in membrane permeability and decrease of MMP, and this loss in MMP is considered as one of the earliest events in the apoptotic cascade [51, 52]. Schapira et al. found that complex I activity reduced in substantia nigra homogenates from PD brain and demonstrated mitochondrial dysfunction participated in PD [53]. Our data showed that when resveratrol was present in the media, the MMP reduction induced by 6-OHDA was significantly alleviated. These findings indicated that resveratrol could confront oxidative stress, increase mitochondrial function, and block 6-OHDA-induced cell apoptosis.

## 5. Conclusion

In summary, resveratrol possesses neuroprotection against 6-OHDA induced damage on PC12 cell. Resveratrol prevented MMP reduction, increased neuronal viability, and attenuated 6-OHDA-induced neuron apoptosis. Furthermore, CXCR4 signaling pathway might participate in this neuroprotective effect of resveratrol. Therefore, the current study could provide new insights into the neuroprotective mechanisms of resveratrol, which also indicated that resveratrol might be a potential therapeutic treatment for neurodegenerative diseases.
